# Different Nursing Care Methods for Prevention of Keratopathy Among Intensive Care Unit Patients

**DOI:** 10.5539/gjhs.v8n7p212

**Published:** 2015-12-16

**Authors:** Reza Pourmirza Kalhori, Sohrab Ehsani, Farid Daneshgar, Hossein Ashtarian, Mansour Rezaei

**Affiliations:** 1 School of Paramedical, Kermanshah University of Medical Sciences, Kermanshah, Iran; 2 Farabi Hospital, Kermanshah University of Medical Sciences, Kermanshah, Iran; 3 School of Medicine, Ophthalmology Department, Kermanshah University of Medical Sciences, Kermanshah, Iran; 4 School of Public Heath, Health Education Department, Kermanshah University of Medical Sciences, Kermanshah, Iran; 5 School of Public Heath, Biostatistics Department, Kermanshah University of Medical Sciences, Kermanshah, Iran

**Keywords:** nursing, intensive care unit, keratopathy, Kermanshah

## Abstract

**Background:**

Patients with reduced consciousness level suffer from eye protection disorder and Keratopathy. This study was conducted to compare effect of three eye care techniques in prevention of keratopathy in the patients hospitalized in intensive care unit of Kermanshah.

**Methods:**

This clinical trial was conducted in 2013 with sample size of 96 persons in three random groups. Routine care included washing of eyes with normal saline and three eye care methods were conducted with poly ethylene cover, liposic ointment, and artificial tear drop randomly on one eye of each sample and a comparison was made with the opposite eye as the control. Eyes were controlled for 5 days in terms of keratopathy. Data collection instrument was keratopathy severity index. Data statistical analysis was performed with SPSS-16 software and chi-squared test, Fisher’s exact test, ANOVA and Kruskal–Wallis one-way analysis of variance.

**Findings:**

The use of poly ethylene cover (0.59±0.665) was significantly more effective in prevention of keratopathy than other methods (P=0.001). There was no statistically significant difference between two care interventions of liposic ointment and artificial tear drop (P=0.844) but the results indicated the more effective liposic ointment (1.13±0.751) than the artificial tear drop (1.59±0.875) in prevention of corneal abrasion (P<0.001).

**Conclusion:**

Results of the study suggest the use of poly ethylene cover as a non-aggressive and non-pharmaceutical nursing and therapeutic method for prevention of keratopathy in the patient hospitalized in intensive care unit.

## 1. Introduction

Vision sense is one of the most important and vital sense of human and human acquires most of his information and findings through this sense (Brito et al., 2010). In a healthy person, eyelids are physical barriers against trauma and dryness of eyes and exposure of pathogenic organisms on the eye surface. Tear keeps eye surface humid and washes microorganisms on the eye surface with the antimicrobial materials (Kocacal Guler, Eser, & Egrilmez, 2011). The patients hospitalized in intensive care unit are exposed to increased risk of eye disorders by removing natural protective mechanisms for eyes such as reduction of tear production and direct corneal reflex. These patients are not able to nictitate and close their eyelid due to reduction of consciousness level and receiving tranquilizers and anesthetic medicines and as a result, risk of eye injuries such as dryness, abrasion, tear, and keratitis will increase (Werli-Alvarenga, Ercole, Botoni, Oliveira, & Chianca, 2011). These risks can range from keratitis to vision loss (Kocacal Guler et al., 2011). In the conducted studies, it was found that eye care of the patients hospitalized in the intensive care unit is ignored considering severity of hazardous and critical conditions and complex and different therapeutic measures which are taken for them (Hernandez & Mannis, 1997; McHugh Alexander, Kalhoro, & Ionides, 2008). Keratopathy or microbial keratitis is a hazardous potential complication resulting from keratitis and can progress rapidly in case of no care and treatment and lead to keratitis within 48 hours (Ezra et al., 2009). Rosenberg (2008) in a study mentioned that 20 to 42% of the patients in intensive care unit suffered from keratopathy and there is lagophthalmos in 75% of these patients (Rosenberg & Eisen, 2008).

Dowson et al. (2005) also showed that different methods were used for taking care of eyes in different centers (Dawson, 2005). In the conducted studies, different eye care methods have been studied in intensive care unit. The study by Hua Shan and Dyomin suggested the more effective use of polyethylene coatings than the use of artificial tear drops in 2010 (Shan & Min, 2010). In the study by Hang Myoco et al. (2008) in China, no statistical difference was reported between the use of cover poly ethylene and Lanolin eye ointment in prevention of keratitis in patients hospitalized in the intensive care unit (So et al., 2008). Guler et al. (2011) also showed that all patients who had used the poly ethylene cover didn’t suffer from keratopathy (Kocacal Guler et al., 2011). In another research, the researchers concluded that there was no statistical difference between two methods of eye ointment and poly ethylene cover in prevention of keratitis (Koroloff et al., 2004). Considering high importance of eye cares in intensive care unit, this comparative study was conducted on three methods of eye care including the use of liposic ointment (carbomer, 980), poly ethylene cover and artificial team drop in prevention of keratopathy in intensive care unit.

## 2. Materials and Methods

This research was a Single Blind Clinical Trial which was registered after receiving agreement of the research and ethics committee of Kermanshah University of Medical Sciences under code IRCT2014010214333N15 in Iran clinical trial site. After necessary correspondences with Imam Reza Educational and Therapeutic Center and coordination with technical authority of the intensive care unit of Imam Reza Hospital about the use and prescription of liposic ointment (carbomer 980) and artificial tear drop, field work started. The inclusion criteria included GCS ≤8, age of above 18 and below 75 years, the absence of direct trauma (lack of midface fracture (1 to 4), intubation of patient and enjoyment of supportive mechanical ventilation, lack of direct corneal reflex, initial corneal health (confirmed by the ophthalmologist with Fluorescein drop and after examination with portable slit lamp), lack of cataract surgery and record of glaucoma disease with open or closed angle, lack of Intracranial pressure (Cushing’s triad including reduction of heartbeat, hypertension and reduction of respiratory rate), informed consent of the legal custodian of patient and no hospitalization in a recent month in intensive care unit. On the contrary, in case of increase in the consciousness level and return of direct corneal reflex (increase of consciousness level to 2 to 10 times per minute), need for cardiopulmonary resuscitation, incidence of intracranial pressure, extubation of patient and dissatisfaction of legal custodian of patients with continuation of treatment, the patient was excluded from the study.

After complete training of three eye care methods to nurses of ICU during an initial examination with slit lamp and staining of fluorescein, corneal surface of the qualified patients was studied. In case of negative Fluorescein test and soundness of corneal surface, the patient was included in the study. Each of the eyes of the patient received one of the eye care methods randomly for 5 days. In the care receiving group with artificial tear drop, two drops were administered in eyes of patient every 2 hours. In the care receiving group with poly ethylene cover, care was taken by covering eye with a piece of polyethylene film from eyebrow to cheek bone with closed eyes and the related cover was changed every 12 hours. In the care receiving group with liposic ointment (carbomer 980), the ointment was put in edge of eyelid in the lower forinx part every 5 hours to cover eye surface and then the eye was closed with hand. Ophthalmologist or internist examined the patients in terms of eye surface disorders and creation of keratopathy without being aware of the eye care method every day for 5 days with slit lamp and fluorescein staining of patients. After making interventions from the first or second day of hospitalization and examining eyes of the patients by the ophthalmologist or internist, the results were evaluated with severity level table of eye surface disorders (Table 1). Demographic information was also collected with information collection form which had been made by the researchers and the criteria for completion of interventions and inclusion in data analysis stage included elapse of 5 days from start of eye care intervention or incidence of eye surface disorders during this term. In addition, the patients whose fluorescein test was positive were cured by the ophthalmologist and also the related physician.

**Table 1:** Severity ranking criterion for incidence of eye surface disorders

Eye surface disorders	
Definition	Severity
Lack of contact keratopathy	Rank 0
Incidence of lesion spots (pits resulting from loss of epithelium cells in one third of the lower epithelium layer of cornea	Rank 1
Incidence of pits (small pits) in more than one third of lower epithelium layer of cornea	Rank 2
Incidence of macro-epithelial defects	Rank 3
Turbidity of stroma layer despite epithelial defects of cornea	Rank 4
Incidence of scar in stroma layer	Rank 5
Incidence of microbial keratitis	Rank 6

Severity ranking table of eye surface disorders has external reliability (consistency) of 0.82 and high Cronbach’s Coefficient Alpha with correlation of 0.92 which has been obtained by Rat et al. in a study in 2000(13).

Statistical analysis of the data was done with descriptive –analytical tests (chi-squared test, Fisher’s exact test, and ANOVA and Kruskal–Wallis one-way analysis of variance) in software SPSS-16. For the normal data, ANOVA and Tukey Test were used and for non-normal data, Kruskal–Wallis one-way analysis of variance was used. To study qualitative data, chi-square test was used.

## 3. Findings

The number of the samples which were studied in this research was 107 persons among whom 7 persons died during the study days and other 4 persons were excluded from the study considering increased consciousness level and return of corneal reflex. At the end, 96 persons were under eye care intervention in three groups (the use of liposic ointment, use of artificial tear drop and use of poly ethylene cover. Among them, 43 persons (44.8%) were male and 53 persons (55.2%) were female. Table 3 shows frequency distribution and percent of the studied samples in terms of gender in three care groups. This gender distribution of samples in each of three care groups including liposic ointment group (p=0.080), poly ethylene cover group (p=0.127) and artificial tear drop group (p=0.127) doesn’t show statistically significant difference. Mean age of the participants in this study was 57.8±15.53 years. There was no statistically significant difference between the samples in terms of age (p=0.062). It is necessary to note that there was no difference between the studied groups in terms of the demographic variables.

**Table 2:** Frequency distribution and percent of the studied sample in three groups in terms of gender

Gender	Liposic ointment	poly ethylene cover	Artificial tear drop	Total sum
**Male**	19(59.4%)	12(37.5%)	12(37.5%)	**43(44.8%)**
**Female**	13(40.6%)	20(62.5%)	20(62.55)	**53(55.2%)**
**P_value_**	0.080	0.127	0.127	

**Table 3:** Mean and standard deviation of the studied variables

Kind of intervention	Age		Intervention		Control		P value
		
	Mean	SD	Mean	SD	Mean	SD	
Liposic ointment	54.19	17.03	2.75	0.91	1.13	0.75	0.12
Poly ethylene cover	60.87	13.08	3	1.19	0.59	0.66	0.001
Artificial tear drop	58.56	15.92	3.09	1.37	1.59	0.87	0.23
Non-intervention	57.84	15.53	2.95	1.17	1.1	0.86	0.45

In this study, the studied patients were divided into three classes of cerebral, internal and general surgery patients due to cause of hospitalization of the studied patients. Hospitalization of 55 persons (diagnosis) was due to cerebral and neuronal disease (57.3%), 10 persons were hospitalized due to general surgery (10.4%) and 31 persons were hospitalized due to internal disease (32.3%). There was no statistically significant difference between three groups in terms of cause of hospitalization (Table 2).

Table 3 shows mean and standard deviation of variables. It is evident that mean age of the each intervention group has been specified. In each intervention group, in addition to mean and standard deviation of age, mean and standard deviation of eye surface disorder degree in the intervention group are given in comparison to the control eye in each patient in the same group. On this basis, the intervention group of poly ethylene cover with mean of 0.59, standard deviation of 0.66, the minimum eye surface disorder and artificial tear drop with mean of 1.59 and standard deviation of 0.87 showed the maximum surface disorder. This Table shows that mean degree of eye surface disorder was 1.10 in three intervention groups and 0.86 in standard deviation. On the contrary, total mean degree of surface disorder was 2.95 with standard deviation of 1.17 in the control group. Considering the chi-squared statistical test, there was statistically significant difference between groups and the poly ethylene cover group was more effective than other groups in prevention of keratopathy (p<0.001).

## 4. Discussion and Conclusion

This study investigated and compared three methods of keratopathy prevention. The final results showed that the use of poly ethylene cover group was significantly more effective than other methods in prevention of keratopathy. Although there was no statistically significant difference between liposic ointment group and artificial tear drop group in prevention of keratopathy of patients hospitalized in intensive care unit but this difference was clinically considerable and showed that the use of liposic ointment (carbomer 980) was more effective than the use of artificial tear drop in prevention of keratopathy of patients hospitalized in intensive care unit. In addition, all three methods mentioned above were more effective than the routine method used in the unit i.e. washing of eye with normal saline. Huashan and Diomin et al. in a study conducted in 2010 in China compared three techniques of eye care (artificial tear drop, creation of moisture chamber, poly ethylene cover) in prevention of keratopathy. The obtained results showed that poly ethylene cover and moisture chamber were much more effective than the use of artificial tear drop (Shan & Min, 2010). The Cortese clinical trial study in Australia compared and evaluated two techniques of using cellulose eye drop and Poly Ethylene Covers in prevention of corneal abrasion in patients hospitalized in intensive care unit and concluded about the use of more effective Poly Ethylene Covers(14). Results of the above two researches are in line with the present study.

In the study by Sav in 2008 in China, there was no considerable statistical difference between the use of Poly Ethylene Cover and lanolin eye ointment in prevention of corneal abrasion in patients hospitalized in ICU (10). A result of this research is not in line with results of the present study. In another study which was conducted retrospectively by Güler et al. in 2010 in Turkey, comparison of two eye care methods i.e. the use of Poly Ethylene Cover and the use of carbomer drop (liposic gel) in prevention of corneal abrasion concluded that the use of Poly Ethylene Cover was more effective than the use of carbomer drop (Kocacal Guler et al., 2011). This result is also in line with the resent research. Results of the study by Karla Da et al. (2012) showed that the use of eye ointment was more effective than closing eyes with adhesive (Dal-Bó, Silva, & Sakae, 2012).

Nair named closing of eyes with adhesive and putting eye ointment as two care interventions in prevention of corneal abrasion and there is no significant difference between them in comparative study (Nair & White, 2013). Results of this study are not in line with results of the present research. Sharifi Tabar et al. (2011) compared two methods of using the eye ointment and closing of eyes with adhesive. Although no significant difference was obtained due to low number of the sample but it showed that the use of eye ointment can be more effective than that of closing eyes with adhesive in prevention of the corneal abrasion (Suresh, Mercieca, Morton, & Tullo, 2000). Results of the study by Ezra et al. (2009) indicated the absence of statistically significant difference between two techniques of eye care with lubricant and geliperm dressing (Ezra et al., 2009). Sivasankar et al. (2006) compared a combination of eye ointment method and taping with swimming goggles for creation of moisture chamber and concluded that method of moisture chamber creation with swimming goggles (such as polyethylene cover) has been more effective in prevention of eye surface disorders (Sivasankar et al., 2006). These results are also in line with this study.

Generally, it can be mentioned that washing eyes of patients hospitalized in intensive care unit with normal saline cannot only act as a method for perfect and effective eye care and as mentioned above, the patients receiving this care (control group) showed the highest degree of corneal abrasion. Therefore, it is recommended to use poly ethylene cover as a selective method. At the same time, it is necessary to introduce different and various methods for eye care and prevention of corneal abrasion in intensive care unit but it seems that more researches can help select better and more efficient methods using more precise and sensitive diagnostic tools. One of the limitations of this study is lack of difference in sampling of patients with GCS between 3 and 8 which was beyond control of the samplers. Age distribution of the hospitalized patients which was beyond control of the researcher was one of the limitations of this study. It is recommended to study lower age range of the patients in the future studies and also include more samples in the study. In addition, considering that the studied population was different in this study as the patients hospitalized in ICU GENERAL with different causes of hospitalization (internal, general surgery and neurosurgery) and considering that variety of metabolic disease type limits generalization of results, it is suggested to study them in the future studies with equal cause of hospitalization.
